# Lipid Droplet Biosynthesis Impairment through DGAT2 Inhibition Sensitizes MCF7 Breast Cancer Cells to Radiation

**DOI:** 10.3390/ijms221810102

**Published:** 2021-09-18

**Authors:** Clelia Nisticò, Francesca Pagliari, Emanuela Chiarella, Joana Fernandes Guerreiro, Maria Grazia Marafioti, Ilenia Aversa, Geraldine Genard, Rachel Hanley, Daniel Garcia-Calderón, Heather Mandy Bond, Maria Mesuraca, Luca Tirinato, Maria Francesca Spadea, Joao Carlos Seco

**Affiliations:** 1 Department of Clinical and Experimental Medicine, University “Magna Graecia” of Catanzaro, 88100 Catanzaro, Italy; clelia.nistico@studenti.unicz.it (C.N.); emanuelachiarella@unicz.it (E.C.); mariagrazia.marafioti@gmail.com (M.G.M.); Ilenia.aversa@unicz.it (I.A.); bond@unicz.it (H.M.B.); mes@unicz.it (M.M.); 2 Division of BioMedical Physics in Radiation Oncology, German Cancer Research Center, 69120 Heidelberg, Germany; f.pagliari@dkfz.de (F.P.); joanaguerreiro@ctn.tecnico.ulisboa.pt (J.F.G.); g.genard@dkfz-heidelberg.de (G.G.); r.hanley@dkfz-heidelberg.de (R.H.); daniel.garcia@dkfz-heidelberg.de (D.G.-C.); 3 Centro de Ciências e Tecnologias Nucleares, Instituto Superior Técnico, Universidade de Lisboa, Estrada Nacional 10 (km 1397), 2695-066 Bobadela LRS, Portugal; 4 Department of Physics and Astronomy, Heidelberg University, Im Neuenheimer Feld 227, 69120 Heidelberg, Germany

**Keywords:** breast cancer, cancer stem cells, lipid metabolism, lipid droplet, DGAT2, radiotherapy

## Abstract

Breast cancer is the most frequent cancer in women worldwide and late diagnosis often adversely affects the prognosis of the disease. Radiotherapy is commonly used to treat breast cancer, reducing the risk of recurrence after surgery. However, the eradication of radioresistant cancer cells, including cancer stem cells, remains the main challenge of radiotherapy. Recently, lipid droplets (LDs) have been proposed as functional markers of cancer stem cells, also being involved in increased cell tumorigenicity. LD biogenesis is a multistep process requiring various enzymes, including Diacylglycerol acyltransferase 2 (DGAT2). In this context, we evaluated the effect of PF-06424439, a selective DGAT2 inhibitor, on MCF7 breast cancer cells exposed to X-rays. Our results demonstrated that 72 h of PF-06424439 treatment reduced LD content and inhibited cell migration, without affecting cell proliferation. Interestingly, PF-06424439 pre-treatment followed by radiation was able to enhance radiosensitivity of MCF7 cells. In addition, the combined treatment negatively interfered with lipid metabolism-related genes, as well as with EMT gene expression, and modulated the expression of typical markers associated with the CSC-like phenotype. These findings suggest that PF-06424439 pre-treatment coupled to X-ray exposure might potentiate breast cancer cell radiosensitivity and potentially improve the radiotherapy effectiveness.

## 1. Introduction

Breast cancer represents the most common cancer in women with an incidence of 2.2 million new cases each year, and a mortality rate of 13.6% of all cancer deaths among women, according to the statistics reported by the International Agency for Research on Cancer (IARC) in 2020 [1]. Currently, screening, early diagnosis, and targeted treatments represent the main strategies for improving breast cancer survival rates.

Radiotherapy (RT) is one of the main approaches for breast cancer treatment and it can be used alone or in combination with other treatments such as surgery, chemotherapy, and immunotherapy. In particular, it has been shown that radiation, in combination with surgery, allows for a better tumor control and increases patients’ survival more efficiently in the early stage of the disease [2]. RT works by causing damages to cellular organelles and producing direct and indirect DNA single-strand breaks (SSBs) and/or double strand breaks (DSBs). Indirectly, RT induces DNA lesions through the formation of reactive oxygen species (ROS) and reactive nitrogen species (RNS) [3]. DSBs, the most critical and difficult type of DNA damages to be repaired, are labelled by phosphorylation of histone H2AX on serine 139 (γH2AX). This event, in turn, recruits all the machinery components involved in DNA damage response (DDR), allowing for cell cycle arrest and DNA repair [4]. However, severe and numerous DNA lesions induce cell death, especially in cancer cells which repair DNA damage less efficiently than healthy cells [4].

Currently, it is believed that RT efficacy is strongly impaired by the presence of a small subset of radioresistant (RR) cancer cells, called cancer stem cells (CSCs) or cancer-initiating cells (CICs) [5]. This small fraction of cells is able to self-renew, differentiate, and invade other organs supporting the initiation, progression, maintenance, and tumor recurrence [6].

Breast cancer stem cells (BCSCs) are characterized by a specific panel of molecular markers, such as high CD44^+^/CD24^−/low^ ratio, the expression of ALDH1 [7], CD133 [8], and CD166 [9] markers.

Several studies have shown that ionizing radiations are able to reprogram differentiated breast cancer cells into a more RR CSC-like phenotype, characterized by a higher grade of tumorigenicity and invasiveness, with the ability to switch between epithelial and mesenchymal-like states [10,11].

Metabolic reprogramming is considered as an emerging hallmark of cancer, implicated in the transition from a dormant state to an active proliferating state in order to fulfill the energetic and biosynthetic demand associated with CSC survival and growth [12]. Recent evidence has also shown that alterations in lipid metabolism, such as the upregulation of de novo lipogenesis, lipid droplet (LD) synthesis, lipid desaturation, β-oxidation, and uptake of exogenous fatty acids (FAs), support cancer stemness in several cancer types [6,12,13,14,15].

LDs are spherical organelles physiologically involved in fat storage and membrane biosynthesis as well as in cell signaling, inflammation, pathogen infection [16], and cancer [17,18]. It has been shown that many solid tumors contain high levels of LDs compared with healthy tissues [19,20]. Recently, Abramczyk et al. have demonstrated by Raman micro-spectroscopy that cytoplasmic LD amounts were two times higher in moderately malignant MCF7 breast cancer cells than in non-malignant breast cells, while highly malignant MDA-MB-231 breast cancer displayed an LD content four times higher than the non-malignant cells, supporting the direct correlation between an increase in LD amount and higher cancer aggressiveness [21]. Interestingly, by using Raman spectroscopy, Roman et al. have demonstrated that high doses of X-rays induced LD accumulation and lipid concentration in human prostate adenocarcinoma cells [22].

Furthermore, several studies revealed that high levels of LDs are a distinctive hallmark of cancer tumorigenicity also in breast cancer [6,13,23,24]. Recently, Hershey et al. have shown that the LD amount was directly correlated with cancer stemness in several breast cancer cell lines. In particular, pharmacological inhibition of acetyl-CoA carboxylase (ACC), by 5-(tetradecyloxy)-2-furoic acid, TOFA, induced a reduction in FAs and impaired LD biosynthesis. This, in turn, affected cell proliferation, stemness, and mammosphere formation in almost all cell lines investigated [24]. Taken together, this evidence suggested that a high LD content characterizes a sub-population of BCSCs, which uses LD accumulation and breakdown in order to proliferate, sustain the crosstalk with the tumor microenvironment, promote aggressiveness, and enhance their resistance to therapies [25].

Several enzymes are involved in lipid metabolism and, by consequence, in LD formation and assembly [6]. Among them, Diacylglycerol acyltransferase 2 (DGAT2) represents the rate-limiting enzyme in the Triacylglycerol (TAG) pathway. It catalyzes the terminal step in the synthesis of TAGs, which are main components of LDs, through the esterification of diacylglycerol with fatty acyl-Coenzyme A in the endoplasmic reticulum (ER). Two different DGAT isoenzymes have been identified in mammals and they are encoded by the DGAT1 and DGAT2 genes. DGAT1 is present only on the ER and interacts with exogenous FAs, whereas DGAT2 is located on the ER and LDs and uses mainly endogenous FAs as a substrate [26]. In particular, Hernández-Corbacho et al. have shown that DGAT2 is constitutively activated in the liver, breast, thyroid, prostate, and pancreatic cancers [26] and that MCF7 cells express higher levels of DGAT2 than DGAT1 [27].

In the light of the above, by interfering with LD biosynthesis, we investigated the effects of a DGAT2 inhibitor (PF-06424439) [28,29] in X-ray-irradiated MCF7 breast cancer cells in order to potentially affect breast cancer cell radioresistance.

## 2. Results

**Hypothesis** **1** **(H1).** *Effects of PF-06424439 on viability, cell cycle distribution and cell death in MCF7 cell line.*

**Figure 1:**
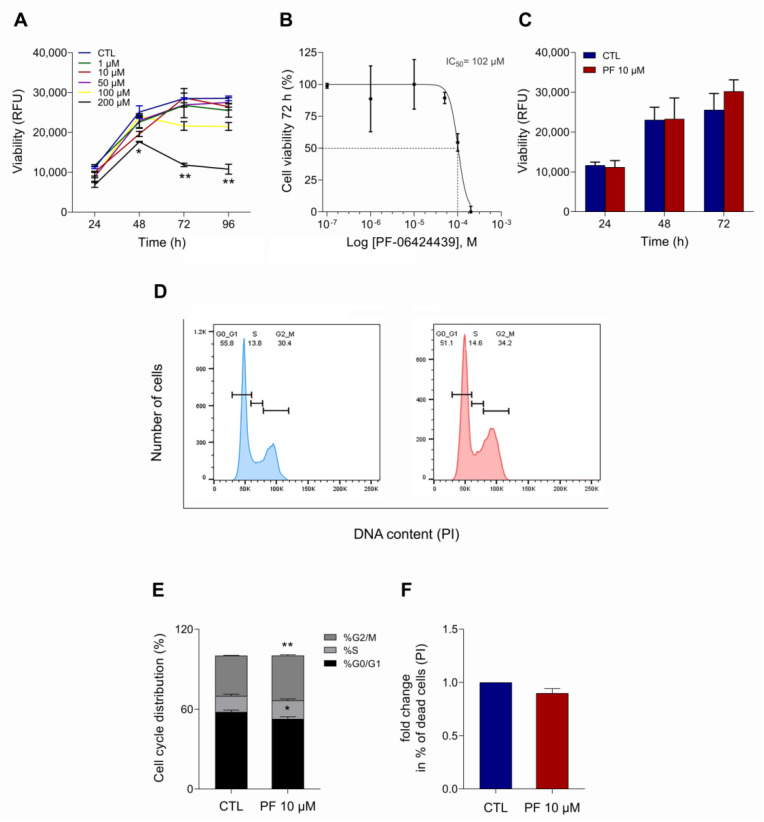
Effects of PF-06424439 on proliferation, cell cycle and cell death of MCF7 cells. (**A**) Cell viability of MCF7 cells treated with different concentrations (1, 10, 50, 100, and 200 µM) of PF-06424439 compared with control (CTL) for 24, 48, 72, 96 h, as assessed by Presto blue assay and measured at 590 nm by using a spectrophotometer. RFU: Relative Fluorescence Units. (**B**) IC_50_ was calculated in response to 72 h of exposure to different concentrations of PF-06424439. (**C**) Cell viability of MCF7 incubated with 10 μM of PF-06424439 for 72 h. (**D**) Cell cycle distribution was analyzed in MCF7 cells treated with 10 μM of PF-06424439 for 72 h compared with untreated cells. (**E**) Quantitative analysis of cells in G1, S and G2/M phases was determined by Flow Cytometry after PI staining as reported in the “Materials and Methods” section. (**F**) The fold change of dead MCF7 cells incubated with 10 μM of PF-06424439 for 72 h was evaluated by Flow Cytometry using PI staining. All data are presented as mean ± SD of three independent experiments for each assay (* *p*  ≤ 0.05, ** *p*  ≤ 0.01).

In order to study DGAT2 role on LD content in MCF7 cells, the effects of PF-06424439 on viability, cell cycle, and cell death were evaluated. MCF7 cells were treated with 1, 10, 50, 100, and 200 µM of PF-06424439 for 24, 48, 72, and 96 h (Figure 1A). Cell viability was inhibited at high concentrations (100 and 200 µM) of PF-06424439 after 48 h, while at concentrations between 1 and 50 µM we observed a progressive cell growth reduction in a dose- and time-dependent manner. The IC_50_ was calculated at 24, 48, 72, and 96 h from PF-06424439 treatment at different concentrations, and the values obtained were, respectively: 214.4 µM, 109.8 μM, 102 μM, and 101.5 μM (Figure 1B). Based on this, MCF7 treatment with 10 μM of PF-06424439 for 72 h was chosen for the following experiments, as it did not significantly exhibit cytotoxic effects on the cells (Figure 1C).

Under these culture conditions, cell cycle analysis revealed a slight decrease in the percentage of cells in G0/G1 phase and the induction of a G2/M peak (Figure 1D). The PF-06424439 effects on cell cycle distribution are also represented in Figure 1E.

Remarkably, no significant differences were observed in the percentage of dead cells when MCF7 were treated with 10 μM of PF-06424439 compared with the control cells (Figure 1F).

**Hypothesis** **2** **(H2).** *PF-06424439 pre-treatment increased the radiosensitivity of MCF7 cell lines via the modulation of lipid droplet content in vitro.*

**Figure 2:**
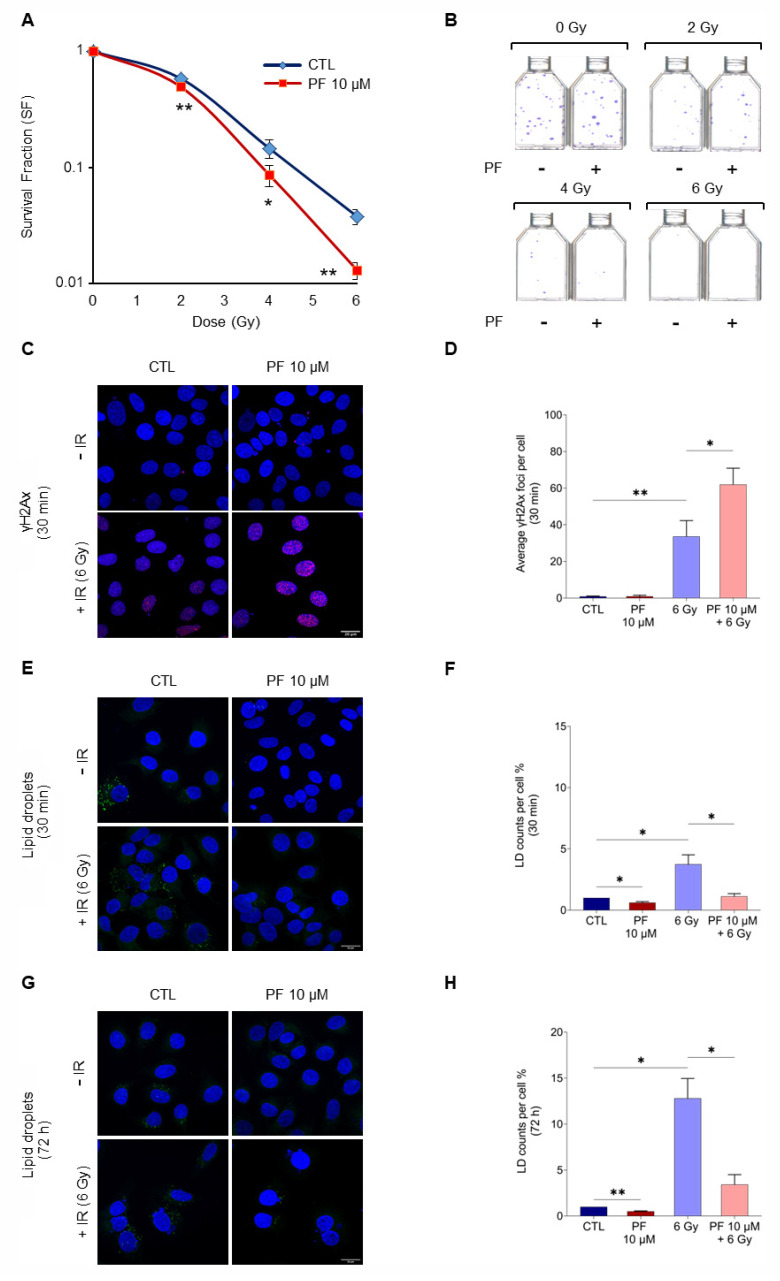
The combined effect of PF-06424439 pre-treatment and radiation enhanced radiosensitivity of MCF7 cells. (**A**) Clonogenic assays were performed to verify the ability of untreated (control, CTL) or PF-06424439 pre-treated MCF7 cells irradiated with different doses of X-rays (2, 4, 6 Gy) to form colonies. (**B**) Representative images of flasks with crystal violet-stained MCF7 colonies after 18 days from 72 h PF-06424439 pre-treatment and radiation (2, 4, 6 Gy). (**C**) γ-H2AX immunostaining was carried out to detect DSBs produced after 30 min from 6 Gy irradiation of MCF7 cells untreated or 72 h pre-treated with PF-06424439. Hoechst 33342 dye was used to counterstain nuclei and 100 cells per sample were imaged at the confocal microscope with a 63× oil-immersion objective. (**D**) The average number of γ H2Ax foci red staining per cell nucleus was shown as bar plot and quantified using Image J 1.52p software. (**E**–**G**) LD540 green staining of cytoplasmic LDs in MCF7 cells untreated or pre-treated with PF-06424439 for 72 h and irradiated with 6 Gy, after 30 min and 72 h from irradiation. 100 cells per sample were acquired with 63× oil-immersion objective. (**F**–**H**) Image J analysis was performed to quantify LD amount at 30 min and 72 h after irradiation. For all confocal images the scale bar is 20 μm. All experiments were performed in triplicate and expressed as mean ± SD (* *p*  ≤ 0.05, ** *p*  ≤ 0.01).

To assess if PF-06424439 treatment could act as an adjuvant in radiation therapy, clonogenic assays were performed and cell survival was measured. MCF7 were pretreated with 10 µM PF-06424439 for 72 h and then exposed to X-rays at different doses (2, 4, and 6 Gy). The combined treatment of PF-06424439 compound with radiation progressively reduced MCF7 cell ability to generate in vitro colonies in an X-ray dose-dependent manner. Colony formation was reduced by almost 65% when pre-treated cells were exposed to 6 Gy compared with irradiated cells without PF-06424439 pretreatment (Figure 2A,B). These data suggested that the use of radiotherapy in combination with PF-06424439 pre-treatment enhanced MCF7 radiosensitivity.

To better investigate the cell response associated with the colony reduction, we carried out experiments aimed at evaluating DNA damage and the altered expression of key lipid metabolism-related genes induced by ionizing radiation.

First, in order to detect DNA double-strand breaks (DSBs) after irradiation, γ-H2AX immunostaining was performed at 30 min (min) after irradiation, which is widely accepted as the time point where the γ-H2AX response is maximal [30,31,32].

Confocal images showed that the induction of DNA damages by 6 Gy irradiation was further stimulated by the 72 h PF-06424439 pre-treatment (Figure 2C). In fact, quantification of γ-H2Ax foci revealed a high number of DSBs in the nuclei of cells subjected to the combined treatment (Figure 2D). This result might, at least partially, contribute to explain the reduced cell survival observed in the clonogenic assays.

Moreover, the combined treatment was also able to modulate the amount of LDs in MCF7 cells. In fact, as shown by confocal microscopy, PF-06424439 pre-treatment significantly decreased LD content compared with the control, while radiation increased LD amount after 30 min and 72 h following 6 Gy X-ray exposure. The 72 h time point after irradiation was chosen because it enabled to select only the most radioresistant cells, that are those surviving to 6 Gy X-rays, and therefore to evaluate the effects of ionizing radiation on LD modulation in this specific cell subpopulation. PF-06424439 pre-treatment significantly reduced the amount of LDs at both time points, compared with the irradiated control (Figure 2E–H).

Oxidative stress was also evaluated in PF-06424439 pre-treated cells shortly after 6 Gy treatment and 72 h post irradiation by staining cells with CM-H_2_DCFDA probe, a general oxidative stress indicator. The results showed that PF-06424439 treatment induced an increase in ROS generation, which was more evident 72 h after IR, both in PF-06424439-treated cells and in combined treatment with 6 Gy X-rays (Supplementary Materials Figure S1).

Taken together, these results highlighted that 72 h-PF-06424439 pre-treatment enhanced radiosensitivity of MCF7 cells after 6 Gy irradiation, exhibiting a higher number of DSBs with a concomitant reduction in LDs and an increase in oxidative stress.

**Hypothesis** **3** **(H3).** *Combined PF-06424439 pre-treatment with radiation influences lipid metabolism and CSC marker gene expression.*

**Figure 3:**
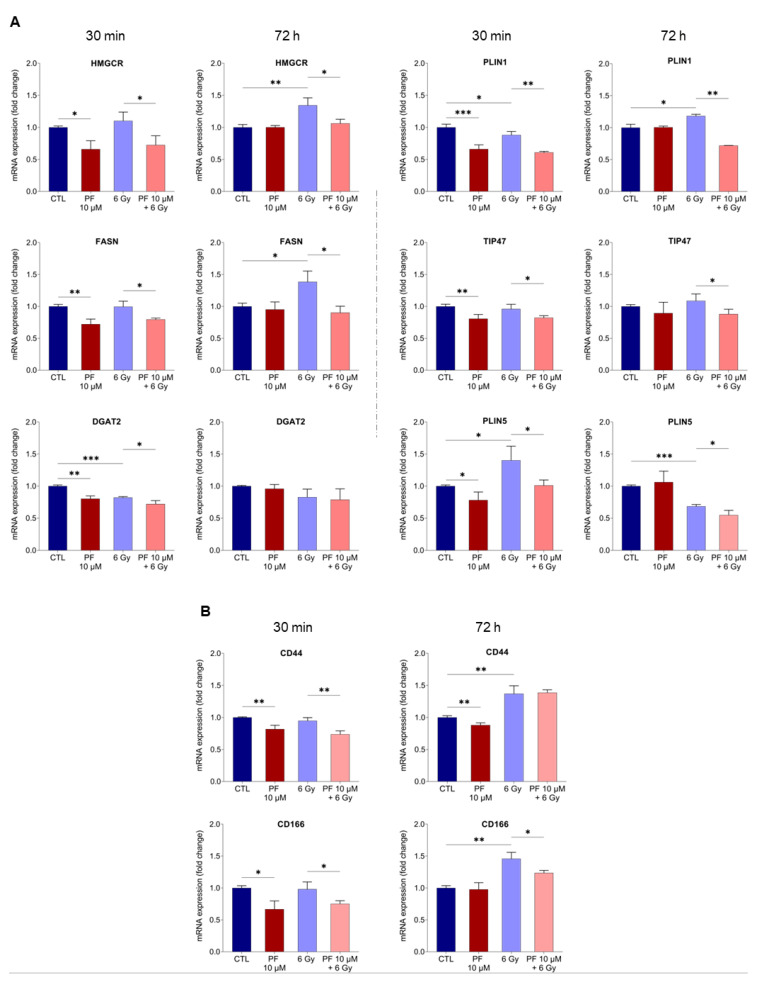
MCF7 pre-treatment with 10 µM PF-06424439 followed by radiation modulates lipid gene metabolism and CSC-related gene expression. (**A**) RT-qPCR results showing the relative mRNA expression of HMGCR, FASN, and DGAT2 as well as LD-associated genes, PLIN1, TIP47, PLIN5 after 30 min and 72 h from irradiation (6 Gy) in MCF7 cells untreated and pre-treated with 10 µM PF-06424439. (**B**) The relative mRNA expression of CSC markers, CD44, and CD166, evaluated by RT q-PCR at 30 min and 72 h after irradiation. Data are represented as means ± SD from three different experiments performed in triplicate (* *p* ≤ 0.05; ** *p* ≤ 0.01; *** *p* ≤ 0.001).

To analyze if the combination of PF-06424439 pre-treatment with 6 Gy X-ray was affecting lipid metabolism, we performed RT q-PCR analysis of lipid metabolism-related genes at 30 min and 72 h after irradiation. The results highlighted that PF-06424439 treatment alone reduced the expression of HMGCR, FASN, and DGAT2, key enzymes involved in lipid metabolism, as well as of LD-associated genes such as PLIN1, TIP47, PLIN5, compared with untreated cells. However, PF-06424439 pre-treatment did not affect the expression of these genes after an additional 72 h from the IR treatment, compared with the untreated cells. Moreover, after 30 min from X-ray exposure, ionizing radiation increased the expression of PLIN5 and reduced the mRNA levels of PLIN1 and DGAT2 compared with the PF-06424439 untreated and unirradiated cells. Nevertheless, at 30 min the combination of PF-06424439 pre-treatment and radiation maintained the expression of the analyzed genes at lower levels in comparison with the just-irradiated control (Figure 3A).

RT q-PCR analysis, after 72 h from irradiation, revealed that X-rays increased the gene expression of HMGCR, FASN, PLIN1 and reduced the gene expression of PLIN5, while the combination PF-06424439-ionizing radiation still reduced HMGCR, FASN, PLIN1, TIP47, PLIN5, compared with the irradiated controls (Figure 3A).

Furthermore, 72 h treatment with PF-06424439 alone decreased the transcript levels of CD44 and CD166, two CSC markers commonly expressed among primary breast carcinomas, compared with untreated cells. However, when analyzed after an additional 72 h without irradiation, only CD44 mRNA levels resulted in a reduction compared with the untreated cells (Figure 3B). Interestingly, the gene expression profile was changed following the combined treatment of PF-06424439 and 6 Gy irradiation. In fact, at 30 min after 6 Gy irradiation and a single 72 h pretreatment with PF-06424439, lower expression of both CD44 and CD166 mRNAs was still observed. However, 72 h post irradiation, while CD44 mRNA levels were stable, a significant reduction was observed for CD166 compared with cells irradiated but not pretreated with PF-06424439 (Figure 3B).

Taken together, although individual gene changes are modest, these data demonstrated that PF-06424439 compound is implicated in modulating lipid metabolism as well as breast CSC marker expression.

**Hypothesis** **4** **(H4).** *PF-06424439 treatment affects epithelial to mesenchymal transition.*

The ability of cancer cells to migrate is associated with tumor invasion and metastasis, both of which are complex phenomena correlated with aggressiveness and poor prognosis.

**Figure 4:**
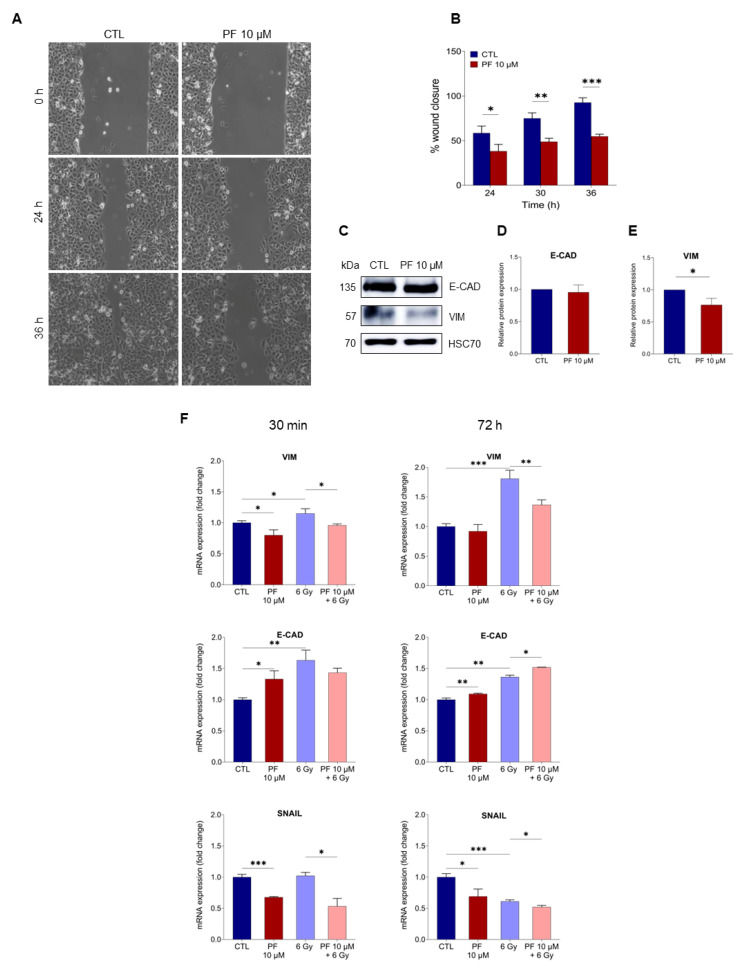
PF-06424439 pre-treatment reduces epithelial to mesenchymal transition on MCF7 cells. (**A**) Migration assay was performed to determine the effects of PF-06424439 on the MCF7 invasive capacity within 36 h from the scratch. (**B**) Migration was significantly decreased in cells pre-treated with PF-06424439; the bar plots show the percentage of wound closure rate from 24 to 36 h, quantified by ImageJ Fiji software. (**C**) Western blotting analysis of Vimentin and E-cadherin protein expression in MCF7 cells analyzed after 72 h of treated 10 µM PF-06424439 treatment. (**D**,**E**) Protein levels were quantified by ImageJ software and the results were normalized to the control. HSC70 was used as a loading control. (**F**) RT q-PCR for EMT markers (E-cadherin, Vimentin, and Snail) of MCF7 cells untreated or pre-treated with PF-06424439 and exposed to 6 Gy, 30 min and 72 h after irradiation. Data were collected from three independent experiments and are shown as means ± SD (* *p* ≤ 0.05; ** *p* ≤ 0.01; *** *p* ≤ 0.001).

Based on the previous results, we investigated the possibility that PF-06424439 pretreatment, by interfering with LD accumulation and survival after irradiation, could influence the migratory abilities of MCF7 cells in vitro. The effect of PF-06424439 on cell migration was evaluated tracking live cells for 36 h under an optical microscope. Wound healing assays demonstrated that PF-06424439 treatment was able to significantly hinder the scratch closure compared with control cells (Figure 4A) with a progressive reduction in cell migration by 20% at 24 h and by 38% at 36 h, compared with the untreated cells (Figure 4B).

The ability to interfere with cell migration was further proved by analysis of protein expression involved in the epithelial-mesenchymal transition (EMT). Western Blot analysis demonstrated that PF-06424439 stimulation induced a clear decrease in Vimentin protein expression as compared with the control. However, no significant difference was found in E-cadherin (E-cad) protein expression (Figure 4C–E). The EMT markers, E-cad, Vimentin, and Snail, were also measured by RT-qPCR (Figure 4F). Although we did not notice any change in E-cad protein level in non-irradiated cells, we observed an increase in the transcript levels for the E-cad gene after 72 h PF-06424439 treatment associated with a reduction in Vimentin and Snail gene expressions. These finding demonstrated that 72 h PF-06424439 treatment was able to interfere with the EMT phenotype. However, after further 72 h without irradiation, Vimentin mRNA level decreased to a similar level as the control, while E-cad and Snail gene expressions still showed a slight increase and a stable reduction, respectively.

Following 6 Gy irradiation, the analysis of the EMT genes revealed that 30 min after irradiation in the 72 h PF-06424439 pretreated cells, the levels of the three genes analyzed remained substantially unvaried, as compared with the PF-06424439-treated and non-irradiated cells. However, ionizing radiation after PF-06424439 stimulation induced a modest but significant decrease in Vimentin and E-cad expression, together with a greater reduction in Snail expression, compared with the irradiated cells (Figure 4F, left panel).

After 72 h following irradiation (Figure 4F, right panel), we observed a significant increase in the transcript levels of Vimentin and E-cad and a reduction in Snail expression in 6 Gy irradiated cells, compared with the unirradiated cells. On the other hand, the combination PF-06424439 pre-treatment and radiation reduced Vimentin and Snail expression, while mRNA levels of E-cad were slightly increased, compared with the irradiated samples.

All together these data demonstrated that PF-06424439 pre-treatment was able to interfere with the ionizing radiation-induced invasive phenotype.

## 3. Discussion

Metabolic reprogramming is currently considered one of the hallmarks of cancer, which is required in order to meet malignancy cell energetic and biosynthetic needs aiming to support their survival and rapid growth [33]. In particular, lipid metabolism alterations, in terms of upregulation of de novo FA and cholesterol synthesis associated with the overexpression of FASN and HMGCR, respectively, and the increased uptake of exogenous FAs and FA β-oxidation (FAO), have been recently recognized to have a crucial role in carcinogenesis [6,12,13,14,23].

Several studies have in fact demonstrated how FA metabolism changes during malignant transformation in breast cancer [34,35,36].

Such lipid alterations very often lead to an increase in cellular LDs that cancer cells use in many ways: as energy storage, source of bioactive lipid mediators derived from arachidonic acid [21], redox homeostasis, protection against ER stress, membrane biogenesis, but also to prevent lipotoxicity from free FAs, DAG, cholesterol, and ceramide by storing them as TAGs, CEs and acylceramides [37,38]. Furthermore, some evidences demonstrated that the LD amount is proportional to the degree of tumorigenicity in breast cancer cell lines [21], sustaining that the aberrant accumulation of these organelles represents one of the main features of BCSCs [24]. Additionally, we have recently shown that in different cancer tissues, including breast, the most radioresistant cancer cells were characterized by a higher LD expression, and that the LD content was tightly regulated by the ferritin heavy chain 1 (FTH1) [39].

Presently, several studies are focused on the selective targeting of the most radioresistant cells combining ionizing radiations with specific drugs. In this regard, lipid pathway inhibitors are gaining a strong interest in this field for their potential role to radiosensitize cancer cells. Lacerada and colleagues have demonstrated that the combination of simvastatin and radiation treatment was able to radiosensitize breast cancer cells. Moreover, a retrospective clinical study highlighted that patients with inflammatory breast cancer and undergoing statin therapy showed a reduction in local cancer recurrence compared with non-statin users when treated with post-mastectomy [40].

One of the enzymes involved in lipid metabolism and LD production is DGAT2, which has been shown to be overexpressed in breast cancer cells [26,27].

In the present study, we report that DGAT2 inhibitor (PF-06424439) pre-treatment promoted radiosensitization of MCF7 breast cancer cells in vitro. First, we demonstrated that 10 µM PF-06424439 72 h pre-treatment did not significantly affect cell viability, but rather cells underwent a cell cycle shift towards the G2/M phase. This indicates that inhibiting the activity of the DGAT2 enzyme induces an arrest of growth with a delayed entry into mitosis without affecting cell survival. Many anticancer drugs arrest cells in the G2/M phase of the cycle and induce cell death and are currently under study as therapeutic approaches for cancer. However, quite often, development of resistance and adverse side effects represent the major limitations for the efficacy of such strategies. Sensitization offers an alternative approach for increasing therapeutic effectiveness while minimizing adverse effects. Indeed, radiation therapy aims at killing clonogenic tumor cells by maximizing the dose delivered to the tumor and reducing the dose received by adjacent healthy tissues. Therefore, sensitizing cancer cells should provide a way to selectively kill the most radioresistant population while better preserving the normal tissues.

We observed a significant reduction in the colony formation potency of PF-06424439 pre-treated MCF7, indicating an effect on the proliferative capacity of a single cell following the combined treatments as compared with irradiation alone. Such a reduction was also associated with an increase in DNA DSBs, an early event occurring soon after irradiation and the extent of which determines the radiosensitivity of cells. This effect was largely additive, as PF-06424439 did not appear either to reduce the clonogenic ability of the cells or to induce DNA DSBs per se. Based on our results, we suggest that such effects could be related to LD modulation. In fact, after a 72 h PF-06424439 treatment, a reduction in LD number was observed, which persisted, at least, for a further 72 h after a single initial administration. Moreover, while 6 Gy X-rays increased LD content in control cells, PF-06424439 pretreatment was able to maintain a reduced LD amount both soon after and 72 h post irradiation.

This was further supported by the mRNA reduced expression of the main structural proteins of LDs, such as PLIN1, TIP47, and PLIN5, after 72 h of PF-06424439 treatment and 30 min after irradiation, as compared with irradiated cells. Moreover, reduced levels of expression for these LD-associated genes were also observed 72 h following the combined treatments, compared with the irradiated cells.

Of note, reduced expressions of FASN (involved in lipogenesis) and HMGCR (involved in cholesterol metabolism) were also observed. This indicates that the inhibition of the DGAT2 enzyme had effects on FA and cholesterol biosynthetic pathways, which are both required for LD homeostasis. On the contrary, radiation significantly increased the expression of PLIN1, HMGCR, and FASN to sustain LD accumulation after irradiation. However, we observed a downregulation of PLIN5 gene expression following irradiation that was fostered by the combined treatments. It has been shown that PLIN5 protects against lipotoxicity mainly in oxidative tissues [6,41], thus the downregulation observed with the combined treatments may indicate a reduced ability of the cells to deal with the oxidative stress, which, in turn, could be implicated in increased radiosensitization. It is worth noting that, as expected, PF-06424439 treatment significantly reduced the transcript levels of DGAT2, which were further decreased by the combined treatment soon after IR. However, although this effect was also produced by X-ray radiation per se, it was associated with LD accumulation, as previously discussed. Moreover, in the RR cell population selected after 72 h from irradiation, DGAT2 expression was not significantly modulated, but was still sufficient to induce detectable changes in genes involved in LD metabolism in the long run.

It has been shown that RR cancer cells can activate several antioxidant pathways in order to reduce ROS generated by radiation therapy. In this context, LDs can play a crucial role to avoid lipotoxicity and retain the cell homeostasis, favoring DNA repair and promoting cell survival [39,42,43,44]. In our conditions, while MCF7 cells were presumably able to deal with the oxidative stress induced shortly after ionizing radiation, they showed increased ROS levels after 72 h of PF-06424439 which resulted further increased 72 h after IR both in cells only pretreated with PF-06424439 and in cells subjected to combined treatments.

Although commonly IR works by increasing ROS production, in this specific situation we were not able to observe any change in basal ROS levels at both time points in non-PF-06424439-treated cells. This might be due to the short lifetime of ROS immediately after IR and/or the enhanced antioxidant systems and detoxification capacity of cancer cells [45,46,47]. Future investigations for a better understanding of this behavior are required.

On the other hand, the prolonged and increased ROS levels observed 72 h after IR in surviving PF-06424439-treated cells might be explained by speculating that the oxidative stress induced by PF-06424439 might further stimulate ROS generation from various cellular compartments, thus leading to an amplified and persistent oxidative stress condition [48]. In this scenario, the reduction in LD content following PF-06424439 treatment might, at least partially, explain for the increased levels of ROS. In fact, LDs are supposed to work also as ROS scavengers [49,50]. Since the majority of the population had lower LDs and resulted arrested in the G2/M phase at the moment of irradiation, it could be that the oxidative stress generated following X-ray treatment was not sufficiently counteracted by LDs and this could contribute to generate the observed high levels of DNA damage. This, in turn, although it remains to be experimentally proven, might hinder the ability of the cells to repair the damages, thus making radioresistant cells less tumorigenic.

Previous studies have shown that after chemo- and radiotherapy, breast cancer cells are enriched in cells with a CSC-like phenotype characterized by resistance to therapies and high CD44 and CD166 expression, two well-recognized cancer stem cell markers [9,51]. To date, the clinical impact of BCSC markers remains unclear. Conflicting results have been reported regarding the correlation between specific markers, the breast cancer stemness phenotype and their prognostic prospective [52]. However, in the present work, we found that PF-06424439 pre-treatment reduced CD44 and CD166 mRNA expression also soon after X-ray exposure. Gene expression profiles analyzed 72 h after irradiation in PF-06424439-pretreated RR cells showed that while there was a not significant upregulation of CD44 mRNA levels compared with the irradiated cells, CD166 mRNA was more sensitive to the combined treatment, showing decreased expression. This suggests that the reduced number of LDs following PF-06424439 pre-treatment could influence the CSC- phenotype in the surviving population, an effect that was preserved also after 6 Gy X-rays and partially maintained 72 h after the X-ray exposure.

Under stress conditions, CSCs have the ability to acquire a more distinct mesenchymal phenotype and to undergo epithelial-mesenchymal transition (EMT), which contributes to tumor cell metastasis and treatment resistance. Indeed, ionizing radiation has been shown to promote these processes, which represent part of the major drawbacks hindering the efficacy of the treatment [53]. Here, we show that PF-06424439 treatment was able to reduce cell migration and the expression of Vimentin protein, which was downregulated also after 72 h from the irradiation in the pre-treated RR cells. Moreover, in these surviving cells, SNAIL and E-cadherin gene expressions were also modulated, indicating that the combination of PF-06424439 pre-treatment with radiation interfered with the migration ability and EMT.

Here, we show that PF-06424439 treatment alone was able to reduce cell migration and the expression of Vimentin mRNA and protein, together with a reduction in SNAIL mRNA and an upregulation of E-Cadherin. This supports the idea that 72 h of PF-06424439 treatment per se was able to reduce the EMT. Soon after irradiation (30 min), in the PF-06424439-treated cells, we observed similar results. After further 72 h, in PF-06424439-pretreated MCF7, the mRNA levels of these genes remained substantially unvaried, except for Vimentin mRNA which returned to control values. This suggests that a single initial PF-06424439 administration could exhibited a long-term effect. Moreover, in the PF-06424439 pre-treated cells surviving to X-ray exposure, a reduction in the EMT was observed if compared with post irradiated RR MCF7. However, in the MCF7 only exposed to 6 Gy, the expression of these EMT markers was not representative of a “typical” EMT phenotype. In fact, the expression of E-Cadherin was increased, and SNAIL levels reduced. Indeed, several studies have reported that the expression of E-cadherin can differently change after irradiation and not always its loss is visible [54]. On the other hand, SNAIL downregulation accounts for the upregulation of E-cadherin, as SNAIL negatively controls E-Cadherin expression [55]. Furthermore, it should be noted that the induction of the hallmark EMT genes is an event triggered at earlier stages than 72 h, thus we cannot rule out the possibility that the gene expressions observed in the RR MCF7 might be influenced by this long time point. Nevertheless, in the present work, after the initial 72 h of PF-06424439, we focused on the cells collected after further 72 h, in order to evaluate the responses of the surviving and more radioresistant subpopulation. In summary, the present study confirms our previous data that X-ray treatment can be a potent LD inducer and provides new evidence that the combined PF-06424439 pre-treatment with radiation is able to enhance radiosensitivity of breast cancer cells, interfering with LD accumulation and clonogenic ability. Our findings imply that the efficiency of radiation therapy might be increased by combined therapies regulating DGAT2 enzymatic activity. Specifically, our results suggest that by preventing high LD accumulation and interfering with lipid metabolism at the moment of radiation exposure might contribute to reduce the radioresistance of cancer cells making the treatments potentially more effective. However, it is worth noting that this study was focused to one breast cancer cell line, whose responses might be cell-type dependent. Therefore, future studies will be oriented to investigate if various breast cancer cell lines with different characteristics and metastatic potentials show similar or different responses, in order to build stronger overall evidence or, alternatively, unravel breast cancer cell type-specific behaviors. Moreover, it will be of huge interest to investigate also the modulations of specific pathways, including lipolysis and fatty acid oxidation, following radiation treatment, in order to find multiple points of intervention.

## 4. Materials and Methods

### 4.1. Cell Culture

Human epithelial breast adenocarcinoma MCF-7 cells (HTB-22) were purchased from the America Type Culture Collection (ATCC^®^, Manassas, VA, USA). Cells were cultivated in DMEM (Thermo Fisher Scientific, Waltham, MA, USA; #11995065) medium supplemented with 10% Fetal Bovine Serum (FBS) (Gibco™, Thermo Fisher Scientific; #10500064) and 1% penicillin/streptomycin (Gibco™, Thermo Fisher Scientific; #15140122) (hereafter referred to as complete medium) at 37 °C and 5% CO_2_ in a humidified atmosphere. Cells were maintained in culture, until they reached 80% confluency, then they were washed with 1× DPBS (Gibco™, Thermo Fisher Scientific; #14190169) and detached by 1× Tryple Express Enzyme (Thermo Fisher Scientific; #12604013).

### 4.2. Pharmacological Treatment

PF-06424439 was obtained from Sigma-Aldrich (Saint Louis, MO, USA, #PZ0233). The powder was dissolved in sterile distilled water to make a stock concentration of 18.65 mM and stored at −20 °C in small aliquots. The effect of PF-06424439 on MCF7 cells was tested at 10 µM by dissolving the appropriate volume of the stock solution in complete medium. PF-06424439 solutions were freshly prepared for each experiment.

### 4.3. Cell Viability Assay

Cell viability was determined using PrestoBlue Reagent 10× (Thermo Fisher Scientific; #A13262). MCF7 cells were seeded at 4.7 × 10^3^ cells/well in 96-well black polystyrene microplates (Corning, NY, USA; #353376) and incubated overnight at 37 °C to allow for cell attachment. The next day, cells were treated with 1, 10, 50, 100, 200 μM of PF-06424439 for 24, 48, 72, and 96 h. At each time point, cells were washed with HBSS (Gibco, Thermo Fisher Scientific; #14025-050) and incubated in the dark at 37 °C with 10 μL of Presto Blue Cell Viability Reagent diluted in 90 μL of fresh complete medium for 90 min. Cell viability was measured at 560 nm (excitation) and 590 nm (emission) using a CLARIOstar^®^ microplate reader (BMG LABTECH GmbH, Offenburg, Germany). Anti-proliferative activity of PF-06424439 was calculated as 50% inhibitory concentration (IC_50_) at 72 h and curve-fitting was performed by non-linear regression analysis (log(inhibitor) vs. normalized response) by using GraphPad Prism 9 software.

### 4.4. Cell Cycle Analysis

Cell cycle distribution in MCF7 exposed to 10 μM of PF-06424439 was investigated by flow cytometric analysis of propidium iodide (PI)-stained cells. Briefly, MCF7 cells were plated in 100 mm dishes at 8 × 10^5^ cells/mL. After 72 h exposure to PF-06424439, 1 × 10^6^ cells/sample were harvested and washed twice with cold DPBS. Cells were detached, spinned down and fixed dropwise with 70% ice cold ethanol (EtOH) in PBS while gently vortexing, then stored at 4 °C for 24 h. Subsequently, samples were centrifuged and incubated with 110 μg/mL of RNase A (Sigma-Aldrich; #R6148) for 30 min at room temperature. The samples were washed twice with DPBS and stained with 20 μg/mL propidium iodide (Sigma-Aldrich; #P4864) for 15 min in the dark. Cells were acquired by means of a FACS Canto™ II (BD Biosciences, Frankin Lakes, NJ, USA) and analyzed by FlowJo software 8.1.

### 4.5. Cell Death Detection

Cell death analysis [56] was performed using a Propidium Iodide (PI) (Invitrogen, Thermo Fisher Scientific). Briefly, 8 × 10^5^ cells were seeded in 100 mm dishes, incubated overnight at 37 °C and then treated with 10 μM of PF-06424439 for 72 h. Subsequently, cells and supernatants were collected, washed twice with cold DPBS, centrifuged and re-suspended in 1 μL of the 100 μg/mL PI working solution. For each sample, an unstained control was produced. Cell suspensions were acquired using a FACS Canto ™ II flow cytometer and analyzed by FlowJo software 8.1. The percentage of Propidium-positive cells was evaluated and plotted as fold change compared with the control.

### 4.6. Clonogenic Survival Assay

Colony forming assay was performed to analyze the ability of MCF7 cells to survive and form colonies after irradiation. To this purpose, 1.1 × 10^6^ MCF7 cells were seeded in triplicate in T75 flasks, exposed to 10 μM of PF-06424439 for 72 h and then irradiated with 2, 4, and 6 Gy, using the MultiRad225 (Faxitron Biotics, Tucson AZ, USA). Subsequently, cells were collected and plated in six replicas at densities proportional to the increasing dose of X-rays in T25 flasks (200, 250, 500, 1000 cells per flask), for performing clonogenic assays. After 18 days, colonies were fixed with 100% EtOH, stained with Crystal violet and manually counted, using 50 cells per colony as threshold. The survival fractions obtained were normalized to the respective unirradiated control sample.

### 4.7. γ-H2AX Staining

To detect DNA double-strand breaks (DSBs) produced after X-ray irradiation, γ-H2AX staining was performed. MCF7 cells were seeded in 6-well plates at a density of 1.4 × 10^5^ cells/well on coverslips and treated with 10 μM of PF-06424439 for 72 h. Afterwards, PF-06424439- treated and -untreated cells were irradiated with 0 and 6 Gy and incubated at 37 °C for 30 min with fresh complete medium. Cells were then washed two times with DPBS and fixed with cold 4% paraformaldehyde (PFA; Pierce 16% Formaldehyde in DPBS, Thermo Fisher Scientific; #28908) for 15 min at room temperature. After two washes, cells were permeabilized with cold 0.25% Triton-X 100 (Th Geyer, Renningen, Germany; #8013) for 5 min and then incubated with 500 μL/well of cold 4% bovine serum albumin (BSA; Sigma-Aldrich; cA6003-25G) for 45 min. Cells were rinsed three times with 1% BSA washing buffer and immunostained with the primary antibody Anti-phospho-Histone H2AX Ser13 (Merck, Sigma-Aldrich; #05-636) diluted 1:1000 in the same buffer at room temperature for 1 h. After washing three times, cells were incubated with Alexa Fluor 647 conjugated anti-mouse antibody (Thermo Fisher Scientific; #A-21236) diluted 1:1000 in washing buffer for 30 min. Samples were rinsed three times with cold 1% BSA and then nuclei were stained with 1 μg/mL of Hoechst 33342 dye (Thermo Fisher Scientific; #62249) at room temperature for 20 min. Afterwards, all the samples were mounted on glass slides using SlowFade Diamond Antifade Mountant (Invitrogen, Thermo Fisher Scientific; #S36972). Images were acquired at the confocal microscope (Zeiss LSM 710) with 63× oil-immersion objective and ImageJ 1.52p software was used to quantify DBSs comparing the samples to MCF7 untreated and unirradiated cells.

### 4.8. Lipid Droplets Staining

Lipid droplet formation in response to PF-06424439 pre-treatment and X-ray irradiation was detected using the lipophilic dye LD540 [14]. Cells were cultivated in 6-well plates at a density of 1.4 × 10^5^ cells/ well on coverslips, treated with 10 μM of PF-06424439 for 72 h and then irradiated with 0 and 6 Gy. After 30 min and 72 h from irradiation, cells were fixed with 4% PFA for 45 min at room temperature. Cells were rinsed twice with cold DPBS and then stained with LD540 diluted 1:5000 in DPBS, in the dark. The cells were then washed two times and counterstained with Hoechst 33342 at a final concentration of 1 μg/mL for 20 min. Two further washes with DPBS were carried out and then coverslips were mounted on glass slides using SlowFade Diamond Antifade Mountant. Zeiss LSM710 confocal-laser-scanning microscope with 63× oil immersion objective was used to detect LDs in all the samples. ImageJ 1.52p software was used for processing the confocal images in Z-stacks and counting LDs.

### 4.9. ROS Staining

ROS levels were measured by freshly prepared chloromethyl dichlorodihydrofluorescein diacetate dye, CM-H_2_DCFDA (Thermo Fisher Scientific; # C6827). After treatments, 4 × 10^5^ cells were washed and stained with 5 μM CM-H_2_DCFDA solution in HBSS for 20 min at 37 °C in the dark. Subsequently, cells were washed, resuspended in PBS, and acquired at a FACS Canto™ II flow cytometer and analyzed by FlowJo software 8.1.

### 4.10. RNA Extraction and cDNA Synthesis

1.1 × 10^6^ MCF7 cells were seeded in T75 flasks, treated with 10 μM of PF-06424439 for 72 h and irradiated with 6 Gy X-rays. After 30 min, 1 × 10^6^ MCF7 cells were collected, while the same amount of cells/sample were seeded again in fresh medium and 1 × 10^6^ cells/sample were collected after 72 h from irradiation. RNA extraction from 1 × 10^6^ MCF7 cells was performed according to the instructions of the High Pure RNA isolation kit (Roche, Basel, Switzerland; #11828665001). Total RNA was resuspended in 50 μL of RNAse-free water and measured on a Nanodrop ND-1000 (Thermo Fisher Scientific). For cDNA synthesis, 1 μg of total RNA was reverse transcribed using the RT2 first strand kit (Qiagen, Hilden, Germany; #330404) according to the manufacturer’s instructions and suspended with nuclease-free water (Thermo Fisher Scientific; #15815408) in a final solution of 200 µL (5 ng/μL).

### 4.11. Q-RT-PCR Analysis

RT-qPCR reactions were carried out using a Power SYBR^®^ Green PCR Master Mix (Thermo Fisher Scientific, Waltham, MA, USA) as described in Guerreiro et al. [57] and analyzed using ABI 7900HT Real-Time PCR System (RT-qPCR) (Thermo Fisher Scientific). Primer sequences are listed in Table 1. For amplification, the temperature profile used consists of one cycle at 95 °C for 10 min followed by 40 cycles at 95 °C for 15 s and 60 °C for 1 m. All measurements were performed in triplicate and each sample was normalized to the stable housekeeping gene β-Actin. The relative gene expression was calculated using the ∆∆Ct method.

### 4.12. Migration Assay

The effect of PF-06424439 on MCF7 cell migration was investigated using wound healing assay. Briefly, 1.1 × 10^6^ cells were seeded in T75 flasks and incubated with 10 µM of PF-06424439 for 72 h. Subsequently, 2.8 × 10^4^ PF-untreated and -treated cells were seeded into culture-insert 4-well plates (Ibidi, Grafelfing, Germany; #80466) and incubated overnight at 37 °C. Afterwards, inserts were removed, cells were washed gently with 1× HBSS and cultured with fresh complete medium. The migration of cells was followed and imaged every 6 h at 10× Magnification for a total of 36 h using an Eclipse Ts2 optical microscope (Nikon). The gap size was measured using ImageJ 1.52p software. In order to evaluate the cell migration ability of cells, gaps’ areas at 24, 30, and 36 h post-wounding were subtracted from gaps’ areas at 0 h and the percentages of wound closure obtained were normalized to the control.

### 4.13. Protein Extraction and Western-Blot Analysis

MCF7 cells were plated in T75 flasks at the density of 1.1 × 10^6^ cells/flask and then treated with 10 μM of PF-06424439 for 72 h. Afterwards, cells were washed twice with cold DPBS and incubated for 20 min with 300 μL of 1× Ripa Buffer (Cell Signaling, Danvers, Massachusetts, USA; #9806S) supplemented with HaltTM Protease Inhibitor Single-Use Cocktail, (Thermo Fisher Scientific; #78425) and HaltTM Phosphatase Inhibitor Single-Use Cocktail (Thermo Fisher Scientific; #78428), both diluted 1:100. Cells were then transferred to microtubes and, after centrifugation at 14,000× *g* at 4 °C for 20 min, the supernatant was collected. Protein concentration was measured by BCA Protein assay kit (Thermo Fisher Scientific; #23227) at 562 nm using BSA to produce a standard curve. For protein analysis, 15 μg of whole cell extracts for each sample were electrophoresed under reducing condition in 10% SDS-polyacrylamide gels and then electrophoretically transferred onto PVDF membrane filters (Bio-Rad Laboratories, Hercules, CA, USA; #1620177), using Trans-Blot Turbo Transfer System (Bio-Rad Laboratories; #1704150). In order to prevent the non-specific antibody binding, blots were blocked for 1 h with BSA blocking buffer, 5% in PBS, with 0.1% Tween-20 (Bio-Rad Laboratories; #1706606). Membranes were washed with PBS-0.1% Tween and incubated with antibodies in blocking solution overnight at 4 °C. Antibodies used were E-cadherin (1:2000) (Cell Signaling; #3195), Vimentin (1:1000) (Cell Signaling; #5741) and Hsc70 (1:2000) (Santa Cruz Biotechnology, Delaware Ave, CA, USA; #sc-7298). PBS-0.1%Tween-20 was used to remove the excess of primary antibodies and then the membranes were incubated in blocking solution with horseradish peroxidase-conjugated secondary antibodies, Goat anti-Rabbit IgG (H+L)-HRP (1:2500) (Thermo Fisher Scientific; #31462) for E-cadherin and Vimentin and mouse IgG BP-HRP (1:2500) (Santa Cruz Biotechnology; #sc-516102) for Hsc70 for 1 h at room temperature. Subsequently, blots were rinsed with 0.1% PBS-Tween and developed with Clarity Western ECL Substrate (Bio-Rad Laboratories; #1705061) using Amersham Imager 680. Protein levels were analyzed by ImageJ 1.52p software.

### 4.14. Statistical Analysis

The results were shown as mean values +/− SD of three independent experiments performed in triplicate, unless otherwise specified. Data were analyzed with Student’s t-test, using GraphPad Prism 9 and were considered significant at * *p*  ≤ 0.05, ** *p*  ≤  0.01, *** *p*  ≤ 0.001.

## 5. Conclusions

In this work, we evaluated the effect of PF-06424439, a selective DGAT2 inhibitor, on MCF7 breast cancer cells exposed to X-rays. On the basis of our results, we can conclude that PF-06424439 reduced the LD number and the expression of many lipid-related genes. This, in turn, translated to an improvement in breast cancer cell radiosensitivity. Lastly, DGAT2 inhibition also influenced the EMT phenotype, as PF-06414439-treated cells showed a reduced migration ability. This evidence could open up new scenarios for the study and possible treatment of breast cancer.

## Figures and Tables

**Table 1:** List of Primers Used for RT qPCR analysis.

Gene	Forward Primer (5′→3′)	Reverse Primer (5′→3′)
β Actin	TCCCTGGAGAAGAGCTACGA	AGGAAGGAAGGCTGGAAGAG
CD44	GGGTTCATAGAAGGGCACGT	GGGAGGTGTTGGATGTGAGG
CD166	CGATGAGGCAGACGAGATAAG	TAGACGACACCAGCAACAAG
VIM	GATGTTGACAATGCGTCTCTG	TGTTCCTGAATCTGAGCCTG
ECAD	ACAGGAACACAGGAGTCATC	TGTTGCTGTTGTGCTTAACC
SNAIL	TCCAGAGTTTACCTTCCAGC	AGAGTCCCAGATGAGCATTG
HMGCR	TTCGCCGACAGTTACTTTC	CTCACAACAAGCTCCCATC
FASN	CGTGCTGAATGAGGAACAG	GACCGCTTTCTTCTGGATG
DGAT2	GCTACAGGTCATCTCAGTGCTC	GTGAAGTAGAGCACAGCGATGAG
PLIN1	GACAAGGAAGAGTCAGCCCC	GAGAGGGTGTTGGTCAGAGC
TIP 47	CACCATGTTCCGGGACATTG	GCACCTGGTCCTTCACATTG
PLIN5	GATCACTTCCTGCCCATGAC	GCTGTCTCCTCTGATCCTCC

## Data Availability

Data presented in this study are available on request from the corresponding author.

## Supplementary Materials

The following are available online at https://www.mdpi.com/article/10.3390/ijms221810102/s1.
