# Supplementary material for: Lipid Droplet Biosynthesis Impairment through DGAT2 Inhibition Sensitizes MCF7 Breast Cancer Cells to Radiation
Source: Int J Mol Sci. 2021 Sep 18;22(18):10102. doi: 10.3390/ijms221810102 (PMC8466244; doi:10.3390/ijms221810102)
Supplement: Supplementary file 1 [file ijms-22-10102-s001.zip › ijms-1298465-supplementary.pdf]

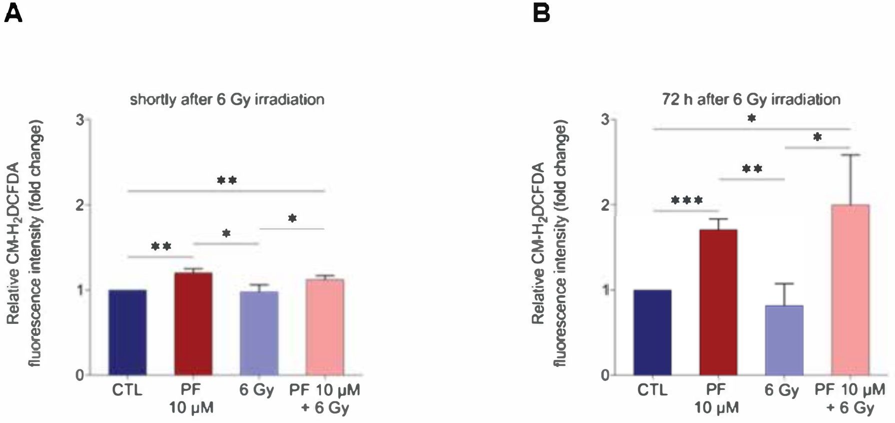

Supplemental Figure S1. Effects of 10 µM PF-06424439 treatment on ROS generation in MCF7 cells shortly after 6 Gy X-rays **(A)** and 72 h post irradiation **(B)**, as assessed by CM-H2DCFDA assay. The results are presented as fold changes compared to control (CTL) of 3 independent experiments ± SD (*p ≤0.05, **p ≤0.01, ***p ≤0.001).
